# Representation of a `positive experience' of surrogacy in Yazd, Iran: A qualitative study

**DOI:** 10.18502/ijrm.v20i9.12067

**Published:** 2022-10-10

**Authors:** Zahra Ghane-Mokhallesouni, Abbas Askari-Nodoushan, Hajieh Bibi Razeghi Nasrabad, Ahmad Kalateh Sadati, Razieh Dehghani Firouzabadi

**Affiliations:** ^1^Department of Social Sciences, Yazd University, Yazd, Iran.; ^2^National Institute for Population Research (NIPR), Tehran, Iran.; ^3^Research and Clinical Centre for Infertility, Yazd Reproductive Sciences Institute, Shahid Sadoughi University of Medical Sciences, Yazd, Iran.

**Keywords:** Surrogate mothers, Emotions, Personal satisfaction, Infertility.

## Abstract

**Background:**

The social and cultural challenges facing surrogate mothers have been explored in several studies. However, few studies have discussed the motivations of surrogate mothers, their expressions and interpretations of their lived experiences, and their feelings of personal and spiritual satisfaction.

**Objective:**

This study aims to present the positive experiences of surrogate mothers from a phenomenological perspective.

**Materials and Methods:**

Using a phenomenological approach, this study was conducted from September 2020 to January 2021 in the city of Yazd, Iran. Participant observation and semi-structured interviews were used to collect the data among 12 participants with at least 1 experience of surrogate motherhood.

**Results:**

Our findings showed that, despite having had harsh physical and socio-cultural experiences such as fear of social labeling and stigma, participants felt a kind of inner satisfaction and a positive view of their actions. A core theme found in the study was mothers' satisfaction. The main categories included feminine self-sacrifice and positive rewards. Feminine self-sacrifice included 2 sub-categories: creating happiness and conveying motherly feelings, while positive rewards included good childbirth, family acceptance, and halal income.

**Conclusion:**

This study showed that surrogate mothers experience conflicting feelings of inner satisfaction and social stigma during surrogacy. Some of those interviewed were willing to go through surrogacy again, but they feared social labeling and stigma, being misunderstood by others who are not fully informed about surrogacy, and being subjected to family and social disapproval.

## 1. Introduction

Family is highly valued in Iranian society and the Islamic religion, and the birth of a child plays an important role in strengthening family bonds (1). Both religious and cultural norms and values reinforce such perceptions. That is why Muslim countries, from Morocco to Iran and other Middle Eastern nations can be described as family-oriented communities in which marriage and childbearing are highly valued (2). One of the factors that can weaken the foundation of the family is infertility. In many families, infertility is a major problem that can lead to stressful experiences and serious psychological problems (3).

In recent decades, many successful attempts have been made to treat infertility using assisted reproductive techniques (ART) such as surrogacy. The absence of a congenital uterus, failure of repeated treatments, recurrent miscarriages, heart diseases, cancer, or abnormal uterine structure are among the reasons for deciding to use this technology (4). ART pose many epistemological and ethical challenges (5, 6). They can lead to emotional effects, physical and financial stress, as well as many moral and legal problems (7, 8). Surrogacy is no exception and is a social and cultural lived experience. The development of the embryo can influence the surrogate mother, in particular their perceptions, feelings, and experiences which are not unrelated to the meanings and concepts produced in the mother's social and cultural world.

Several studies have reported that returning the child to the requesting couple after birth can cause emotional distress in the surrogate mother (7, 9, 10). For those women who leave the baby, there may be a risk of postpartum depression, as well as feelings of anger or guilt (11). However, some studies have reported that most surrogate mothers have a positive experience of surrogacy and do not suffer the psychological consequences of carrying a fetus (12). From a psychological point of view, they do not have problems or discomfort in delivering the child, and most of them have a morally meaningful or positive experience of this action (13–15). With about 2-3 million infertile couples, Iran is the only Muslim country where ART have been legitimized by the religious authorities (15, 16). This has given Iran a unique position in the Islamic world (13). Currently, 61 infertility clinics (24 public and 37 private) operate in several major cities of Iran such as Tehran, Isfahan, Shiraz, Tabriz, Yazd and Mashhad (16). Although previous studies have examined the feelings and motivations of these mothers, there is still a research gap to explore surrogacy within the socio-cultural context of Iran (5, 12, 13). For example, few studies have discussed the motivations of surrogate mothers, their interpretations of their lived experience, and their feelings of personal and spiritual satisfaction.

Therefore, this paper aims to present the positive experiences of surrogate mothers from a phenomenological perspective. Yazd province was chosen for the setting of this research as it was the location of one of the first specialized infertility treatment centers in Iran. Conducting social and cultural research on surrogacy can provide an opportunity to deepen our understanding of the consequences and processes of women experiencing surrogacy.

## 2. Materials and Methods

This qualitative ethnographic study was conducted at the Yazd Reproductive Sciences Institute, Yazd, Iran to assess the experiences of surrogate mothers. We used a phenomenological anthropological methodology. The study was conducted in a period of 5 months (September 2020 to January 2021). The participants were 12 women who had been referred to the Yazd Reproductive Sciences Institute, Yazd, Iran to act as surrogate mothers. The inclusion criterion was that the surrogate mothers had experienced surrogacy at least once and no more than 7 yr passed from the time of their surrogacy. Participant observation and semi-structured interviews were employed for the data collection. The researchers wanted to understand the experiences of surrogate mothers from an anthropological and cultural phenomenological perspective. In phenomenological research, it is necessary to consider the concrete social phenomena, conditions and events (17). In this regard, it can be said that surrogacy should be studied not as an axiom and independent of nature, but as a social structure. If we want to study the issue in-depth, we must use a phenomenological method and field study as well as participant observation.

As the name suggests, to collect data and provide valid interpretation through participant observation, the researcher takes the role of a participant in the social life of the people in the place of interest and in relation to the issue(s) being studied. The purpose of participant observation is to study social behaviors, not artificially or in a laboratory, but in a real and natural environment (18, 19).

In this study, one of the researchers initially sat for hours in the research institute as a member of the community and monitored all the behaviors of the clients. The observations were carried out anonymously so that clients would show their normal behaviors. Sometimes the researcher would show up as a client with infertility herself to be able to communicate more easily and intimately with surrogate mothers. In total, about 50 hr of participant observation were collected to gain a fuller understanding of what the interviewees were saying. In addition, in several cases, the researcher visited the participants with their permission to interview them and examined their living conditions more closely. In addition to participant observation, semi-structured interviews were used. The interviews were conducted with 12 participants (Table I).

Each interview lasted from 2.5-3 hr, and the researcher allowed the surrogate mothers to speak on any topic within the frame of the study's research questions. The interview would begin with questions such as: Can you tell me how you came to know about surrogacy? Can you tell me why you decided to be a surrogate mother? May i know more about your experiences? Are you satisfied with it now?The order of the questions was not important and depended on the interview process. The researcher tried to interview in a quiet and safe environment. All interviews were recorded on a cell phone and then transcribed.

The 7-step Colaizzi method was used to analyze the data (20). At first, the participants' interviews were read and re-read several times so that the researchers could gain a deep understanding of the contents of the interviews. Then the important statements related to each phenomenon were extracted and the meaning of each statement was also extracted. In the next step, the obtained meanings were classified into clusters of themes. The researcher then obtained a detailed and complete description of the phenomenon. In addition, the researcher tried to provide a network of concepts and themes about the experience of the mothers participating in the research. The last stage of the Colaizzi's method which was carried out was to present the participants with the findings to test the validity of the research; the researchers were able to further determine the reliability of the data through discussing the research findings with the medical staff at the institute to consider the truth or falsity of the data given the staff's judgment, based on their long experience dealing with surrogate mothers (15).

In the present study credibility, transferability, confirmability, authenticity, and dependability were considered during the process of data collection and data analysis in order to promote the reliability of findings.

**Table 1:** Characteristics of the participants


**Code**	**Age (yr)**	**Gestational age (yr)**	**Residence**	**Marital status**	**Number of children**
**1**	42	38	Yazd	Widow	2
**2**	35	35	A village near Yazd	Divorced	1
**3**	32	31	A village near Yazd	Married	1
**4**	32	32	Bandar Abbas	Married	3
5*	37	34	Esfahan	Divorced	1
**6**	35	35	Yazd	Divorced	1
**7**	35	35	Mashad	Married	4
**8**	31	31	Kerman	Married	2
**9**	27	27	Yazd	Divorced	1
**10**	30	27	A village near Yazd	Divorced	1
**11**	42	36	Yazd	Married	2
**12**	33	33	Herat	Divorced	2
*This participant became a surrogate for her sister. Other participants had no kinship relationship with the infertile couples					

### Ethical considerations

In this research, ethical issues such as conscious consent, privacy, anonymity and confidentiality were considered. Interviewees were reassured that their statements would not cause them any problems. For the study, only those who were completely satisfied with the interview conditions were interviewed. To maintain anonymity, the names of the interviewees were not mentioned in the paper, and to comply with confidentiality, the participants' private information was not presented here. This study was approved by the Ethical Committee of Yazd University, Yazd, Iran (Code: IR.YAZD.REC.1398.031).

## 3. Results

Most participants in the study had experienced problems such as social rejection of surrogacy by people around them as well as family issues such as poverty. In addition, some participants mentioned physiological and physical problems and associated pain and suffering. The surrogate mothers in this study reported numerous problems and complications they had during pregnancy both physically and mentally. However, since the physical consequences of surrogacy were not the focus of this article, they are only briefly mentioned as follows. They referred to problems like long back pain, weakness and lethargy, headaches, as well as complications from cesarean section. One of the participants had the experience of repeated surrogacy and decided not to be a surrogate mother again due to other physical complications. However, some participants considered these consequences to be natural effects of pregnancy and still wanted the procedure to be repeated. They referred to frequent pregnancies in the older generation and therefore focused less on the physical consequences of their decision, and considered these to be an inherent part of pregnancy.

Despite this, the results indicated that the participants were generally very satisfied and pleased with their motherhood experience. They considered the experience a divine success and grace. This is contrary to many other studies on surrogacy which have focused on its negative consequences on the mothers and their motives. Based on our results, the surrogate mothers' actions could be divided into 2 categories: a) feminine self-sacrifice, and b) positive reward, which is divided into several sub-categories (Table II).

### Feminine sacrifice

All participants showed a strong desire to make the lives of other families happy. Although their own lives might have been full of sorrow and grief, they were very happy that they were giving a gift to another family. They said that it was enough for them to give hope to a family, and to open a new window in their lives. It was the feeling of a mother or a woman who felt happy about herself by helping others and enjoying it because she can give joy and smiles to others.

This belief can arise from the religious and cultural conditions of the community, which can make the women feel good by helping others. Surrogacy has 2 consequences: on the one hand, their financial needs are met and on the other hand, they satisfy the needs of another person. They feel they are doing something important and valuable. In their view, enabling another person to experience motherhood who otherwise would not be able to do so is highly valued in Islam and the Iranian family-oriented culture. Surrogate mothers are very kind and compassionate. They feel good that they convey a motherly feeling to a woman to make her happy.

According to this theme, a woman's only attachment is to her child. When she cannot have a child of her own, she will be ashamed and sad. However, once she receives this sweet motherly feeling, she will wish that all women can experience this feeling. Surrogate mothers volunteer to pass this feeling on to others. In other words, she is the mediating link for the transmission of this feeling. Because the surrogate women are mothers themselves, they understand the feelings of an infertile woman. Therefore, they try to help every woman feel motherhood and enjoy it at least once. Mothers say that no woman on earth should be deprived of this beautiful feeling.

### Positive reward

Surrogate mothers know that there is both sadness and joy in the path they have taken. On one hand, pregnancy can be very troublesome and difficult for a woman. On the other hand, they experience the feeling of motherhood during surrogacy and are able to pass on this feeling to another, making an infertile woman happy. Mothers consider the cultural dimension of this work more important and are encouraged to volunteer. They use positive justification to facilitate this by saying that they do not have much difficulty during pregnancy and give birth easily. Although there is no easy pregnancy for any mother, perhaps this kind of encounter with the phenomenon is a kind of self-satisfaction for these mothers to convince themselves to become more willing to accept surrogacy. They pay attention to the human and spiritual dimension of this practice, although undoubtedly there are always exceptions.

Some surrogate mothers prefer that their children know the truth about the matter so that they may better understand and help them. But in some cases where the children are young and cannot understand the truth, the mothers often hide it from them. They prefer not to let their children know and say that their upcoming child has been aborted so that it is both easier for them and their family. This secret will be kept in the family and will not be accidentally revealed.

Some children who can understand the truth can be informed so that they can help the mother at home. These children take care of the mothers at home and support them along their way. Furthermore, the surrogate mother who has the support of her husband and has received his approval peacefully has more support during pregnancy, and pregnancy problems become easier for her. In some cases, the husband's support even goes beyond that of his own children and he becomes a special guardian of the child as he considers that child a commitment to which he has strongly pledged. He feels responsible for anything about the baby and tries to prevent any problems during the 9 months.

The issue of halal income is of special importance to surrogate mothers. They believe that income from surrogacy is halal. They try to observe all the moral and legal principles of the rights of the child (sometimes even more than their biological children). This commitment is rooted in the socio-cultural context of Yazd, where deep-rooted religious values emphasize the importance of earning a halal livelihood (13).

Surrogate mothers in our field study believed that the money from the rental womb is very blessed and sacred. They believe that because infertile couples willingly pay this money to them to sustain their married life, this money has wonderfully Barekat (blessing). Some also believe that because they make couples happy, this income makes them happy and changes their life. The lives of these mothers are often not very good and sometimes they even start their lives completely from scratch thanks to this income, which can lead them to success and wellbeing.

It is believed that when a mother's heart is happy and she wishes well from the bottom of her heart, it is enough for this world and the hereafter. These women say they can prosper day by day and deliver righteous children to society because they believe that if a child grows up with legitimate money, he/she will surely become a righteous person.

However, there are exceptions to the above, and different views were also recorded in this study. Respondent number 3 said:

“After all, I am a mother and I would like to give them a good child. For 2 yr now, I have been looking for a family that is both financially supportive and happy with my help to them, and I can experience the feeling of being a mother.” (This shows that she was eager to make others happy and to transfer the feeling of motherhood to others.)

An observation report stated:

“A surrogate mother is ready to go to the operation room to implant the fetus. The infertile mother is standing next to her and is very worried. She keeps rubbing her hands together, worried that she might not get a positive response this time again because this was the second time they were attempting a transfer. But the surrogate mother, even though she is worried and full of stress tells her: Do not worry so much, trust in God. Hopefully, it will be fine this time and you will have a baby”.

Another observation report stated:

“The interviewer was talking to the woman when the son came home from work (A boy about 27-28 yr old). He greeted as soon as he arrived and stood in front of his mother and said: Feel better mom? Did you have a headache today? Did you take your diabetes pill?” It was clear from the conversation between the mother and the child that there was a good relationship between them, and her mother also said: My son is very careful about me during pregnancy. He's worried. God bless him”.

**Table 2:** Categories related to the satisfaction of surrogate mothers


**Category **		**Condensed meaning unit**	**Example participant quotes**
**Feminine sacrifice**			
	Rejoicing others	Participant 7: “That person has faith in me and i try to make them happy”
	**Pleasing others**	Being full of sorrow, but rejoicing in the happiness of others	Participant 12: “I'm glad i gave life to someone else, but i hurt myself”
		The desire to convey this feeling to others	Participant 4: “I want to transfer the feeling of motherhood to another. You know, when you become a mother, you no longer belong to yourself”
	**Transfer of** ** motherhood feeling**	Giving motherhood feeling to someone who cannot otherwise be a mother	Participant 11: “Because i am a mother myself, i wanted someone else to feel it too. There is a difference between someone who has a child and who does not”
**Positive reward**			
	Justification to make surrogacy problems easier	Participant 4: “I have easy pregnancy and delivery”
	**Easy pregnancy**	Relieving pregnancy problems	Participant 3: “Fortunately, i bear very easily”
		Informing the older child, as he/she can understand it, and hiding it from other children	Participant 8: “My elder son knows it, but my younger does not. He thinks i had an abortion”
	Children's support of the mother during pregnancy	Participant 7: “For the first 3 months i had varicose veins, and my kids were very affectionate because they had accepted that we had no other choice”
	**Family acceptance**	Husband's support	Participant 9: “My husband takes care of the child as our child. He says that this is a religious duty”
		Divine reward	Participant 9: “If you just solve one problem for someone, God will solve hundreds of problems in your life”
		Halal business	Participant 2: “It is very important for me that everything is halal and haram in life. When i inquired, i was relieved and told myself that if it was not halal, the court would not approve it
		**Halal income**	Receiving wages as halal act	Participant 11: “I was told that they wanted it to be halal, and it is my right to receive payment for it. I did not say anything at all”

## 4. Discussion

Surrogacy is one of the technologies that is now available in many parts of the world, including Iran. However, further research is needed to study the numerous and important positive and negative dimensions and consequences of these technologies in different social and cultural contexts. For example, the multiplicity of mothers for a child can lead to confusion in defining and forming the individual and familial identities of the child in the future (2, 21). In addition, surrogacy can challenge the long-standing characteristics of the Iranian family, which have long been based on a strong biological relationship between the mother and child. This technology has also moved pregnancy, once considered a private matter in the relationship, into the realm of the public and medical spheres (22, 23).

Of course, the positive dimension of this technology should not be ignored. The use of this technology can be a way to prevent divorce and strengthen the foundation of the family (24). Women who have dreamed of becoming mothers and have never become mothers are now hopeful with this technology. In contrast, a mother who could not even afford to buy bread for her child can now support her family and not be ashamed before her child thanks to the income from surrogacy.

As can be seen from the analysis of the interviews, the surrogate mothers felt satisfied with what they had done and even interpreted it as divine merit. The satisfaction of surrogate mothers has also been reported in other studies, which have found that women who perform surrogacy have described this process as a satisfying experience, as many of the surrogate mothers in these studies expressed the experience of uterine donation in words such as “increased self-confidence” or “increased self-respect” (25, 26). There are also opposing views, for example which argue that surrogacy abuses women and creates female exploitation, helping men to dominate women (6, 27).

This study investigated the reasons why surrogate mothers are happy with surrogacy. Surrogate mothers gain new strength to continue life as they increase their self-confidence and hope. They gain an altruistic feeling that they can make another person happy and in return earn a halal income that is approved by society. Many observers believe that the commercial surrogate mother is contrary to medical ethics (26). In the present study, the majority of respondents received a salary in addition to payment for the relevant costs.

According to the results, economic motives in deciding to rent a womb with the aim of earning money, gaining personal and social independence, and not relying economically on others were of great importance for surrogate mothers. In most cases, the financial motivation was dominant, and in some cases an emotional bond between the child and the mother was formed during and after pregnancy. The findings of this study, consistent with previous research (13), showed that even for those women who made financially motivated decisions, other reasons may have played a part with varying degrees of importance, such as empathy, lack of social support, reward in the hereafter, and a sense of commitment they had toward caring for the fetus. As other historical and cultural studies conducted in our research site have pointed out (28, 29), part of this sense of commitment and spiritualism stems from the historical culture of Yazd, belief in God and deep-rooted religiosity in Yazd, which emphasizes the work ethic as well as the importance of earning a halal income based on Islamic rules. Therefore, participants were happy and satisfied with the fact that they earn halal income in accordance with Islamic rules.

Usually there was a formal agreement between the infertile couples requesting a child and the surrogate mothers, according to which the surrogate mother becomes pregnant with the fetus belonging to the infertile couple and must take care of it until the end of the pregnancy and deliver the child to her genetic parents after delivery. Nevertheless, infertility treatment centers are generally not aware of this agreement and its provisions, because legislation has not yet regulated this matter. Therefore, the required payment amount has not been determined and there is a lot of disagreement about the nature of this type of contract and its provisions (8). However, according to the findings of this study and other studies conducted in Iran, infertile couples and surrogate mothers generally have formal or informal contracts or agreements between themselves and determine the payment amount themselves. For example, in 1 recent study, the average 9-month income of surrogate mothers was 50 million Tomans (30). Therefore, it is necessary for the legislator to define the legal provisions for such contracts. Doing so will put an end to differences of opinion as well as assign tasks to the applicant's families and medical centers.

The findings showed that the surrogate mothers considered themselves empowered in society because they have done a great job and given birth to a child for an infertile woman. The surrogate mothers gained this sense of inner satisfaction through perceptions of divine justifications and good deeds. They have a dual sense of perspectives. On the one hand, they face many problems. They may not be willing to be recognized in society as a surrogate since this practice is not also welcomed in society. On the other hand, they associate their work with spiritual intentions, and in this way, they get rid of fears, sorrows and challenges, and feel inner satisfaction. They gain a sense of forgiveness, helpfulness, and hopefulness by giving a motherly feeling to others and fulfilling the wishes of others, creating an objective and tangible justification for themselves. Finally, we believe that the participants were trying to build a different understanding of surrogacy in a connection between new technology and the socio-cultural concepts of Iranian society and specifically Islamic values. They tried to highlight the spiritual dimensions of their work by invoking Islamic concepts. According to our results, this happened through the connection between maternal and emotional concepts on the one hand, and spiritual concepts of Islam on the other. However, they did also experience physical and physiological problems.

## 5. Conclusion

The present study showed that surrogate mothers are happy with their decision for economic, religious and altruistic reasons. Some of them even tended to do it several times. The authors believe that surrogacy should not be used as a way to earn money, as women's health may be compromised through surrogacy.

Surrogacy, like any other technology, has both positive and negative social consequences, and in addition to its positive experiences, which were studied in the present paper, it can also lead to social, psychological, health, and many other negative consequences for those involved. In this study, we focused only on the motivations of surrogate mothers, their interpretations of their lived experience, and their feelings of personal and spiritual satisfaction. Thus, we tried to show how the surrogate mother positively justifies her decision and represents her motivations. Numerous challenges and negative consequences of surrogacy have been mentioned in different social contexts by previous studies and more innovative research is still needed on the subject.

##  Conflict of Interest

The authors declare that there is no conflict of interest.
